# Editorial: Education and health as social determinants: the econeurobiology of brain development

**DOI:** 10.3389/fpubh.2024.1488824

**Published:** 2024-09-26

**Authors:** Raed Mualem, Leon Morales-Quezada, Shir Shance, Calixto Machado

**Affiliations:** ^1^Econeurobiology Research Group, Research Authority, Oranim Academic College, Kiryat Tiv'on, Israel; ^2^Department of Physical Medicine and Rehabilitation, Harvard Medical School, Spaulding Rehabilitation Hospital, Boston, MA, United States; ^3^Department of Clinical Neurophysiology, Institute of Neurology and Neurosurgery, The University of the Medical Sciences of Havana, Habana, Cuba

**Keywords:** public health, econeurobiology, brain development, education, health

## Introduction

The development of the human brain is a dynamic and complex process, profoundly influenced by the surrounding environment during childhood. Early life experiences and educational enrichment play a crucial role in brain development, highlighting the interplay between genetic and environmental factors (1). The field of econeurobiology provides an essential framework for understanding how these factors interact to shape neurobiological development. This perspective is particularly significant in recognizing how education and health function as critical social determinants that influence cognitive and behavioral outcomes in children.

This Research Topic of Frontiers in Public Health brings together a collection of studies that explore these interactions in depth, emphasizing the key factors that impact brain development and the long-term effects on children's behavior and academic performance. The research underscores the profound influence of early-life experiences—from the positive effects of supportive educational environments to the harmful consequences adverse childhood experiences (ACE), toxic stress and trauma (2).

By focusing on the concepts of developmental neuroplasticity and brain connectivity, these studies offer valuable insights into the mechanisms by which environmental conditions shape the developing brain. Moreover, the integration of Gardner's multiple intelligences into educational strategies is emphasized as a means to enhance cognitive and emotional resilience (3). Collectively, the articles in this Research Topic provide essential knowledge for educators, policymakers, and healthcare professionals dedicated to fostering optimal development in children.

## Key factors shaping brain development

The studies in this Research Topic demonstrate that the environment plays a crucial role in brain development, significantly influencing cognitive and behavioral outcomes.

Mualem et al. emphasize six critical factors in brain development: a nurturing environment, adequate nutrition, physical activity, music, sleep, and brain connectivity as explained by Gardner's multiple intelligences. The study highlights how these elements promote cognitive and emotional growth, while also noting the detrimental effects of trauma and deprivation on long-term health and learning outcomes.

Tian et al. explore the relationship between life-course household wealth mobility and adolescent health in rural China. Key findings show that upward wealth mobility, especially during early childhood, is associated with better physical growth, cognitive development, and lower behavioral problems, underscoring the critical role of socioeconomic conditions in shaping long-term health and development outcomes.

Sánchez-Ferrer et al. examine the emotional impact of COVID-19 home confinement on children in Spain. Findings indicate that nearly 40% of children experienced poor emotional states, including fear, sadness, and irritability. Factors such as sleep disturbances, lack of outdoor access, and parental anxiety exacerbated these effects, while creative communication and having pets mitigated emotional distress.

Mucignat-Caretta and Soravia review how environmental factors, both positive and negative, influence human brain development. Music training is highlighted as a beneficial factor that enhances cognitive and motor skills through brain plasticity, while stress is shown to negatively impact brain structure and function. The findings underscore the significant role of environmental inputs in shaping cognitive and emotional development.

Liu et al. systematically review the relationship between fundamental movement skills (FMS) and health-related fitness in children and adolescents. They find strong evidence linking FMS with better cardiopulmonary function, muscle strength, and endurance, while also showing a negative correlation with body composition. The review underscores the importance of developing FMS for overall physical health and fitness.

Melby et al. examine the associations between adolescents' physical literacy, sport and exercise participation (SEP), and wellbeing. Findings reveal that higher physical literacy correlates positively with SEP and various aspects of wellbeing, including self-esteem and life satisfaction. These associations are particularly strong among girls, suggesting that physical literacy is crucial for enhancing adolescents' emotional and social wellbeing.

Lapidot et al. investigate the connection between the gut microbiome and cognitive development in school-aged children, finding that greater microbial diversity is positively linked to higher cognitive function as reflected in IQ scores. Recent research highlights how dietary preferences, particularly traditional vs. processed foods, affect cognitive performance and social behavior in kindergarten children, underscoring nutrition's vital role in early development (4). Socioeconomic status also significantly influences gut microbiome composition and cognitive outcomes.

Ba et al. examine the prevalence and determinants of meeting minimum dietary diversity (MDD) among children aged 6–23 months in three sub-Saharan African countries (Gambia, Liberia, and Rwanda). The findings reveal that only 23.2% of children meet MDD, with significant variations by country, socioeconomic status, maternal education, and access to healthcare, highlighting critical disparities in child nutrition.

Elhady et al. identify multiple barriers to providing adequate nutrition care for child malnutrition in a low-resource setting. Key barriers include insufficient training for healthcare providers, a shortage of nutritional supplements, inadequate patient education materials, and systemic issues like workforce shortages. These challenges hinder effective nutrition care, emphasizing the need for targeted improvements in resources, training, and health system management.

Posner and Rothbart discuss how understanding and strengthening brain networks can enhance elementary education. Key insights include the role of brain networks in reading, writing, number processing, attention, and motivation. Strengthening these networks through targeted educational strategies can improve learning outcomes and foster a growth mindset, highlighting the importance of neuroscience-informed teaching practices in early education.

You et al. identify elevated plasma levels of CCL5 as a potential risk factor for developing tic disorders in children. Elevated CCL5, along with other cytokines like PDGF-AA, was significantly associated with tic disorder development, though not with tic severity. These findings suggest CCL5 could serve as a biomarker for predicting the onset of tic disorders.

Finally, in a key article, Mualem et al. illustrate the influence of neural pathways on classroom learning, using the example of story writing. The optimal development of these connections is vital for fostering both quick, intuitive thinking and more deliberate analysis, leading to what is referred to as the “optimized brain.” Additionally, the article introduces the “Econeurobiology of the Brain for Healthy Child Development” model, showing how a child's ecological environment affects neurological development. This interaction shapes cognitive abilities, emotional regulation, and overall wellbeing, underscoring the importance of a supportive and enriching environment for optimal brain development.

## Conclusion

In conclusion, this Research Topic provides a comprehensive exploration of how environmental and social determinants, particularly education and health, play a pivotal role in brain development. The insights offered in these articles underscore the importance of a multidisciplinary approach to fostering optimal cognitive and emotional growth in children, emphasizing the critical need for supportive environments that enhance brain connectivity and overall wellbeing.
